# DNA barcoding reveals cryptic diversity in the underestimated genus *Triplophysa* (Cypriniformes: Cobitidae, Nemacheilinae) from the northeastern Qinghai-Tibet Plateau

**DOI:** 10.1186/s12862-020-01718-0

**Published:** 2020-11-12

**Authors:** Tai Wang, Yan-ping Zhang, Zhuo-yu Yang, Zhe Liu, Yan-yan Du

**Affiliations:** 1 College of Animal Science and Technology, Gansu Agricultural University, Lanzhou, China; 2 Gansu Key Laboratory of Cold Water Fishes Germplasm Resources and Genetics Breeding, Gansu Fisheries Research Institute, Lanzhou, China

**Keywords:** DNA barcode, Qinghai-Tibet, Tibetan loach, Cryptic species

## Abstract

**Background:**

The northeastern part of the Qinghai-Tibet Plateau (QTP) presents a high number of plateau loach species. As one of the three major groups of fishes distributed on the QTP, plateau loach has high ecological value. However, the taxonomy and systematics of these fish are still controversial, and a large number of new species have been reported. The reason for this phenomenon is that the degree of morphological variation is low, the phylogenetic information provided by morphological and anatomical features used for species identification is relatively poor, and many cryptic species are observed. Based on the high-density sampling points from the biodiversity hotspots surveyed, this study aims to evaluate the biodiversity of plateau loach in the northeastern part of the QTP and reveal the hidden diversity by comparing morphological species with molecular operational taxonomic units (MOTUs).

**Results:**

After careful identification and comparison of the morphology and DNA barcoding of 1630 specimens, 22 species were identified, with 20 considered valid local species and two identified as new species that had not been previously described. Based on the combination of morphological and molecular methods, a total of 24 native species were found, two of which were cryptic species: *Triplophysa robusta sp1* and *Triplophysa minxianensis sp1*. Fourteen of the 24 species form clusters of barcodes that allow them to be reliably identified. The remaining cases involved 10 closely related species, including rapidly differentiated species and species that seemed to have experienced incomplete lineage sorting or showed introgressions.

**Conclusions:**

The results highlight the need to combine traditional taxonomies with molecular methods to correctly identify species, especially closely related species, such as the plateau loach. This study provides a basis for protecting the biodiversity of plateau loach.

## Background

With problems such as global climate change and issues related to populations and the ecological environment, energy and food production is becoming increasingly serious; moreover, achieving sustainable anthropogenic development and understanding and meeting the requirements of biodiversity are becoming urgent research issues [9, 36, 44]. There is a major global demand for accurate and rapid identification of species for the protection and sustainable use of biodiversity resources. Species identification and classification is a basic requirement for biological research. Based on morphological characteristics, classical taxonomy has made great contributions to species classification, however, due to morphological plasticity, traditional taxonomy cannot accurately distinguish all species, particularly for similar and related species [54, 61]. Therefore, new methods of supporting species identification with classical taxonomy methods are needed. Tautz et al. [67] first suggested using DNA sequencing, namely, DNA taxonomy, as the main platform for biological classification. Professor Paul Hebert from the University of Guelph in Canada then introduced the concept of DNA barcoding, highlighting its significance to the field of biological taxonomy and species identification [28, 60] and suggesting the use of the mitochondrial cytochrome C oxidase subunit I (COI) gene as the basis for animal DNA barcoding. The applicability of DNA barcoding to the identification of marine and freshwater fish species has been shown by using a short fragment of approximately 650 bp from the mitochondrial COI gene to identify species based on sequence differences [7, 30, 37, 69, 75, 79, 80] . A growing number of studies have shown that DNA barcodes are widely used in animal species identification and classification, cryptic species detection, phylogenetic research, etc. (Smith et al. 2008; Swartz et al. 2008; Rock et al. 2008) [1]. Moreover, DNA barcodes have been used to construct barcode databases, such as the Barcode of Life Data Systems (BOLD) (https://www.boldsystems.org), which barcoded approximately 96,425 fish specimens belonging to 10,267 species. DNA barcoding can complement traditional species identification as a method of automating and standardizing the process of specimen identification, thereby reducing the dependence on the experience of taxonomists [8, 63].

The Qinghai-Tibet Plateau (QTP), known as “the roof of the world”, is rich in biodiversity and a relatively unique area with many endemic species [34]. The native fish living in the Qinghai-Tibet region belong to three orders: Salmoniformes, Siluriformes and Cypriniformes [76]. *Triplophysa*, which belongs to the family Nemacheilinae (Cypriniformes), is widely distributed on the QTP and in its adjacent regions [74] and represents a special group adapted to the climatic characteristics of the QTP, such as cool temperatures and oxygen shortages [76, 84]. In 1992, 33 *Triplophysa* species were identified. However, over time, a large number of new species have been described, and a total of 140 valid species have been identified [27, 40]. Although some synonymous species may occur [27, 55], these studies showed that a large amount of unknown biodiversity exists in the genus *Triplophysa*, and many species have not been recognized or described. The phenomenon in which many new species are identified is caused mainly by the existence of cryptic species or the lack of a careful classification review. The simple body structure and relatively conservative morphological evolution of the plateau loach fish coupled with their weak migration ability due to the restrictions of the water system have led to limited gene exchange between different populations. Although morphologically imperceptible, the process of species differentiation, including genetic structural differentiation and reproductive isolation, may have occurred over time; thus, many hidden taxa may have been ignored. Therefore, the genus *Triplophysa* should be considered in the study of cryptic diversity.

Classical morphological classification has always played a dominant role in species identification, although it has limitations. In particular, for fish of the genus *Triplophysa*, the phenotype is easily affected by biological factors and the external environment, and morphological plasticity is observed; therefore, morphological differences are not easily detected [27]. Moreover, some species were named many years ago and were assigned relatively simple morphological descriptions. All these factors have led to difficulties in the subsequent identification of species and taxonomic research. Due to the difficulty in obtaining detailed data for comparisons, the distribution of some species may be artificially expanded and mistakenly divided into different geographical populations [17, 55]. This long confusing classification history is particularly obvious in the species classification of *Triplophysa*. Fish biodiversity must be identified and recorded to achieve effective species conservation and predict biodiversity responses to climate change [4, 46, 53]. Although DNA barcode technology has certain limitations, such as improper software models or parameters, which lead to excessive division or clustering of species, DNA barcode itself introgression, pseudogenes, definition of molecule as a result [11, 14, 49, 64], DNA barcoding has been proposed as an important tool that can help close the large gap in our current understanding of biological diversity [5, 15, 16, 43]. Some relatively automated methods of species division using DNA barcoding data sets have been developed, and they may increase the efficiency and decrease the subjectiveness of species division [19, 20, 35].

To date, studies have not focused on the identification or evaluation of cryptic biodiversity within the genus *Triplophysa* using DNA barcoding in the northeastern QTP. Herein, based on extensive sample collection in this area, DNA barcoding technology was used to evaluate the biodiversity of plateau loach in the northeastern QTP, which is a hotspot of biodiversity, and generate a DNA barcoding database of plateau loach in this area. The relationships between morphological species and molecular operational taxonomic units (MOTUs) were assessed, and the hidden biodiversity in the genus was identified. This research will contribute to a more comprehensive understanding of plateau loach biodiversity and aid in the protection of this important fish population.

## Results

**Fig. 1:**
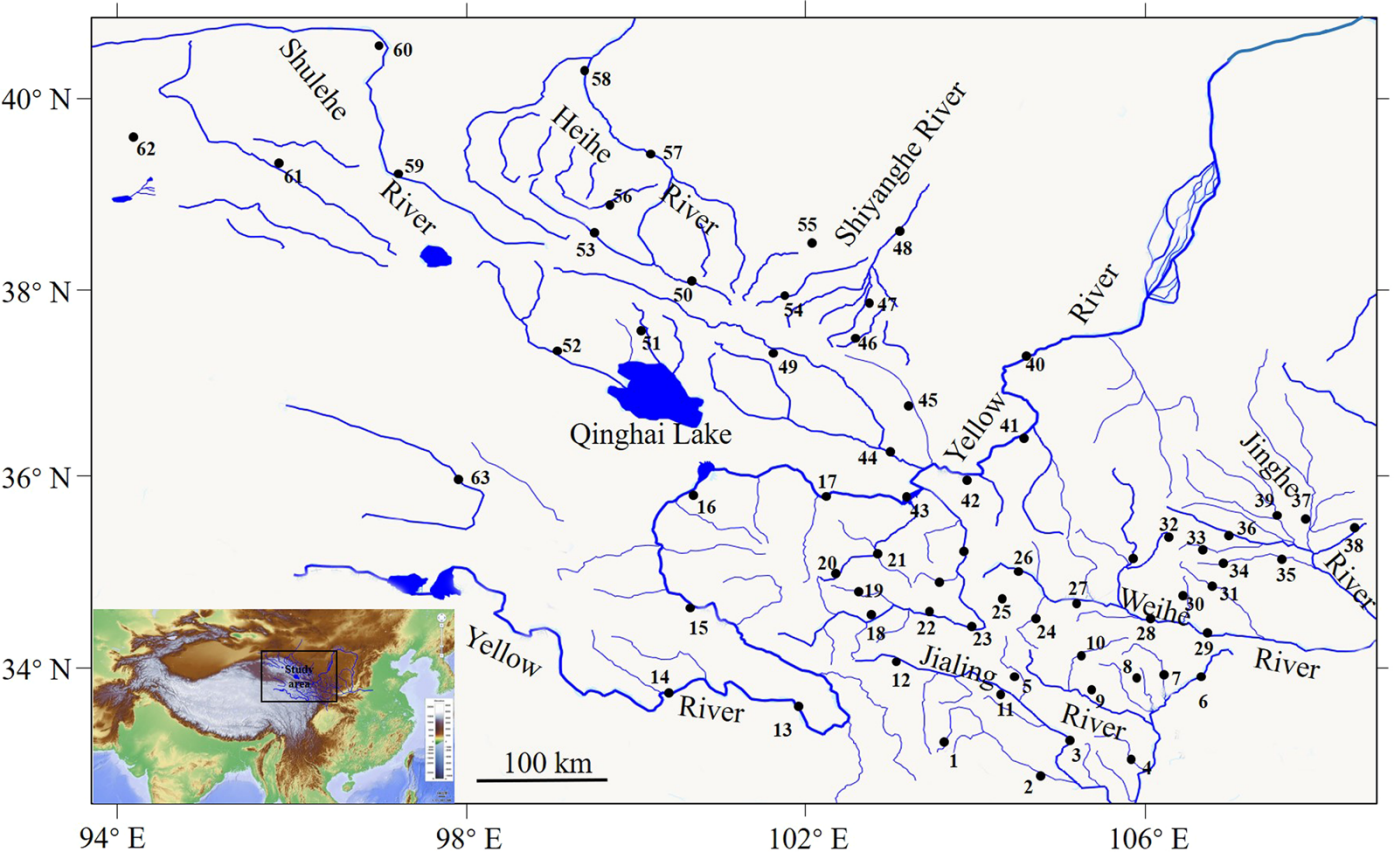
Collection sites. Details of the 111 sites and collected specimens are provided in Additioanl file 1: Table S1. (Sector names of the sampling sites: 1. Jiuzhai; 2. Wenxian; 3. Wudou; 4. Kangxian; 5. Tanchang; 6. Liangdang; 7. Huixian; 8. Chengxian; 9. Xihe; 10. Lixian; 11. Zhouqu; 12. Diebu; 13. Maqu; 14. Hongyuan; 15. Henan; 16. Longyangxia; 17. Jishishan; 18. Luqu; 19. Hezuo; 20. Xiahe; 21. Linxia; 22. Zhuoni; 23. Minxian; 24. Wushan; 25. Zhangxian; 26. Weiyuan; 27. Gangu; 28. Qinzhou; 29. Maiji; 30. Qingshui; 31. Zhangjiachuan; 32. Jingning; 33. Chongxin; 34. Huating; 35. Lingtai; 36. Kongtong; 37. Xifeng; 38. Ningxian; 39. Zhenyuan; 40. Wufo; 41. Pingchuan; 42. Lanzhou; 43. Yongjing; 44. Minhe; 45. Yongdeng; 46. Zhuanglang; 47. Liangzhou; 48. Minqin; 49. Menyuan; 50. Arou; 51. Gangcha; 52. Tianjun; 53. Qilian; 54. Huangcheng; 55. Jinchang; 56. Sunan; 57. Linze; 58. Gaotai; 59. Yumen; 60. Guazhou; 61. Subei; 62. Akesai. This base map was obtained from 91 Vita Assistant software https://www.91weitu.com/index.htm and was edited in Adobe Photoshop CS5 software.)

**Fig. 2:**
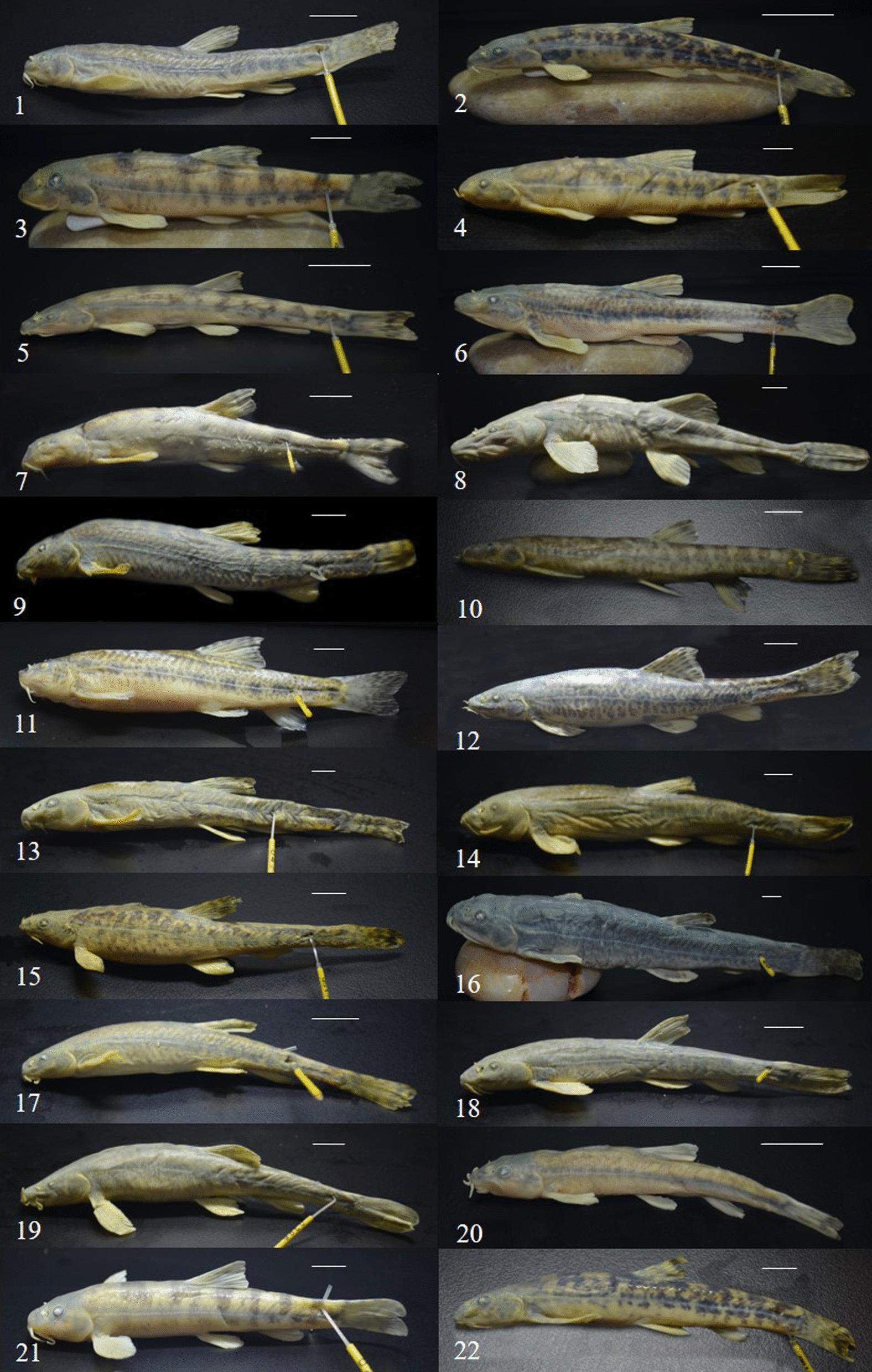
Studied specimens of *Triplophysa*. (1. *T. dalaica* G003; 2. *T. stoliczkai* G0070; 3. *T. polyfasciata* G0187; 4. *T. bleekeri* G0195; 5. *T. robusta* G0531; 6. *T. obscura* G0822; 7. *T. pappenheimi* G0852; 8. *T. siluroides* G0873; 9. *T. hsutschouensis* G0915; 10. *T. minxianensis* GS0213; 11. *T. pseudoscleroptera* GS0216; 12. *T. scleroptera* GS0230; 13. *T. strauchii* GS0273; 14. *T. papilloso-labiatus* GS0305; 15. *T. wuweiensis* GS0381; 16. *T. orientalis* GS0400; 17. *T. shiyangensis* GS0432; 18. *T. leptosoma* GS0441; 19. *T. tenuis* GS0500; 20. *T. sellaefer* GS0560; 21. *T. sp1* GS562; 22. *T. sp2* GS565. Scale bars equal 1 cm.)

A total of 1630 native specimens were collected from the northeastern edge of the QTP (Additional file 1: Table S1; Figs. 1; 2), and 22 morphospecies, including two undetermined species (*Triplophysa sp1* and *Triplophysa sp2*) were identified. Among the specimens, the endemic species *T. robusta* (n = 413) had the largest number of individuals, followed by *T. minxianensis* (n = 253). The undetermined species *T. sp1* (n = 3) and *T. bleekeri* (n = 5) had the lowest number of specimens, with 68 specimens per species on average (Table 1). A total of 1630 COI sequences were obtained. The size of the sequences obtained was 606 bp after trimming to a consensus length. No stop codons were observed, and the mean nucleotide composition within the complete data set was 30.6% thymine (T), 26.7% cytosine (C), 24.3% adenine (A) and 18.4% guanine (G). There were 393 conserved sites, 213 variable sites, 178 parsimonious sites and 35 singleton sites. A total of 230 unique haplotypes were generated in the 1,623 COI sequences. The haplotype number of *T. robusta* was the largest (N*h* = 46), followed by that of *T. obscura* (N*h* = 27) and *T. stoliczkai* (N*h* = 25). The haplotype numbers of *T. bleeker* and *T. orientalis* were the smallest (N*h* = 1). Correspondingly, the haplotype diversity of *T. robusta* was the highest (*h* = 0.9360 ± 0.006). The nucleotide diversity was the highest for *T. obscura* (π = 0.00777 ± 0.00145) (Table 1).

**Table 1:** Sampling information number of individuals and diversity parameters for the specimens included in this study

Species	Collection site (River)	Numbure of specimens (N)	Numbure of haplotypes (Nh)	Haplotype diversity (h)	Nucleotide diversity (π)
*Triplophysa bleekeri* (Sauvage *et* Dabry, 1874)	Jialing River	5	1	–	–
*T. dalaica* (Kessler, 1876)	Jinghe River	55	9	0.469 ± 0.083	0.00114 ± 0.00102
*T. hsutschouensis* (Rendahl, 1933)	Heihe River, Shulehe River, Shiyanghe River	46	8	0.731 ± 0.041	0.00440 ± 0.00125
*T. leptosoma* (Herzenstein, 1888)	Shulehe River	7	3	0.667 ± 0.160	0.00126 ± 0.00095
*T. minxianensis* (Wang et zhu, 1979)	Yellow River, Jinghe River	253	21	0.385 ± 0.040	0.00074 ± 0.00001
*T. minxianensis sp1*	Yellow River, Weihe River	20	2	0.526 ± 0.036	0.00087 ± 0.00047
*T. obscura* (Wang, 1987)	Jialing River, Weihe River, Taohe River	234	27	0.877 ± 0.012	0.00777 ± 0.00145
*T. orientalis* (Herzenstein, 1888)	Taohe River	19	1	–	–
*T. papillosolabiatus* (Kessler, 1879)	Heihe River, Shulehe River	95	13	0.603 ± 0.036	0.00201 ± 0.00112
*T. pappenheimi* (Fang, 1935)	Yellow River, Weihe River	21	4	0.610 ± 0.114	0.00196 ± 0.00011
*T. polyfasciata* (Ding, 1996)	Jialing River	10	3	0.600 ± 0.131	0.00121 ± 0.00082
*T. pseudoscleroptera* (Zhu and Wu 1981)	Yellow River, Xiahe River	9	4	0.583 ± 0.183	0.00138 ± 0.00105
*T. robusta* (Kessler, 1876)	Yellow River, Jialing River, Shiyanghe River	219	46	0.936 ± 0.006	0.00588 ± 0.00182
*T. robusta sp1*	Jinghe River	194	15	0.591 ± 0.020	0.00133 ± 0.00012
*T. scleroptera* (Herzenstein, 1888)	Yellow River, Taohe River	44	3	0.090 ± 0.059	0.00015 ± 0.00005
*T. sellaefer* (Nichols, 1925)	Jinghe River	22	5	0.338 ± 0.128	0.00089 ± 0.00101
*T. shiyangensis* (Zhao & Wang, 1983)	Shiyang River	25	11	0.770 ± 0.086	0.00367 ± 0.00180
*T. siluroides * (Herzenstein, 1888)	Yellow River, Xiahe River, Taohe River	28	5	0.529 ± 0.105	0.00108 ± 0.00095
*T. sp1*	Yellow River, Jialing River	3	2	0.667 ± 0.314	0.00660 ± 0.00269
*T. sp2*	Jialing River	67	6	0.172 ± 0.062	0.00029 ± 0.00007
*T. stoliczkai* (Steindachner, 1866)	Yellow River, Xiahe River, Taohe River, Jinghe River, Jialing River, Shiyanghe River	129	25	0.812 ± 0.028	0.00285 ± 0.00149
*T. strauchii* (Kessler, 1874)	Heihe River	11	2	0.509 ± 0.101	0.00084 ± 0.00056
*T. tenuis* (Day, 1877)	Heihe River, Shulehe River	97	11	0.378 ± 0.061	0.00082 ± 0.00010
*T. wuweiensis* (Li and Chang 1974)	Shiyanghe River	17	3	0.404 ± 0.130	0.00090 ± 0.00085
Total		1630	230		

**Fig. 3:**
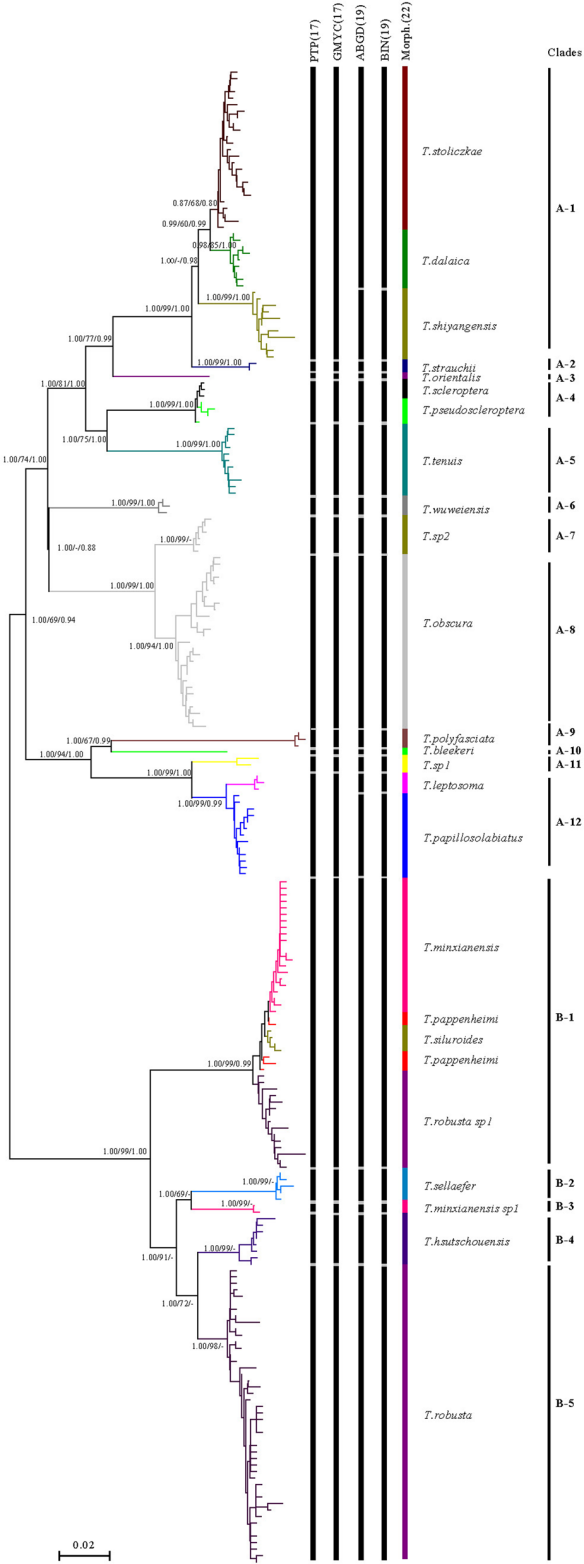
Fifty-percent majority-rule consensus tree showing the clustering of the MOTUs obtained by the four MOTU delimitation algorithms. (The values at the node represent support values in the NJ/ML/BI analysis.) (NJ bootstrap support values below 0.50, ML bootstrap support values below 50 and Bayesian posterior probabilities below 0.50 are not shown.) The length of clade indicates the percentage of divergence. The ruler at the bottom represents a 0.02 replacement for each site. The branch colours match those in the right coloured segmented bar, whose coloured segments represent morphological species. The four black segments bar on the left relative to the species delimitation methods delineate the entities detected by each method. The finer black segments bar on the far right represents the division of the main clades

The phylogenetic tree was constructed by the neighbour-joining (NJ) method, maximum likelihood (ML) method and Bayesian inference (BI) method. With *Homatula variegata* as the outgroup (GenBank no.: MF953219), the topological structure of the phylogenetic trees obtained by the three analysis methods was basically the same. Only the topological structure of the NJ tree was retained here, and the values at the nodes represent the support degree at the nodes of the NJ/ML/BI tree. The Poisson tree processes (PTP) model analysis with an ML partition and Bayesian implementation resulted in 17 MOTUs (Fig. 3). The general mixed Yule-coalescent (GMYC) model analysis identified the same 17 MOTUs obtained in the PTP analysis (likelihood ratio = 76.41, *P* < 0.0001), whereas the Automatic Barcode Gap Discovery (ABGD) and BOLD detected 19 MOTUs for the 22 morphological species (Fig. 3). *T. strauchii*, *T. orientalis*, *T. tenuis*, *T. wuweiensis*, *T. polyfasciata*, *T. bleekeri*, *T. sp1*, *T. sellaefer*, *T. minxianensis sp1*, *T. hsutschouensis* and *T. robusta* showed correspondence between the morphological species and MOTUs. The MOTUs of *T. minxianensis*, *T. pappenheimi*, *T. siluroides*, *T. pappenheimi* and *T. robusta sp1* could not be distinguished by the PTP, GMYC, ABGD or BOLD analysis. The same phenomenon occurred between *T. stoliczkae* and *T. dalaica* and between *T. scleroptera* and *T. pseudoscleroptera*. *T. leptosome* and *T. papilloso-labiatus* could not be distinguished by the PTP or GMYC analysis, although they could be distinguished by the ABGD and BOLD analyses. The same findings were observed for *T. shiyangensis*.

**Fig. 4:**
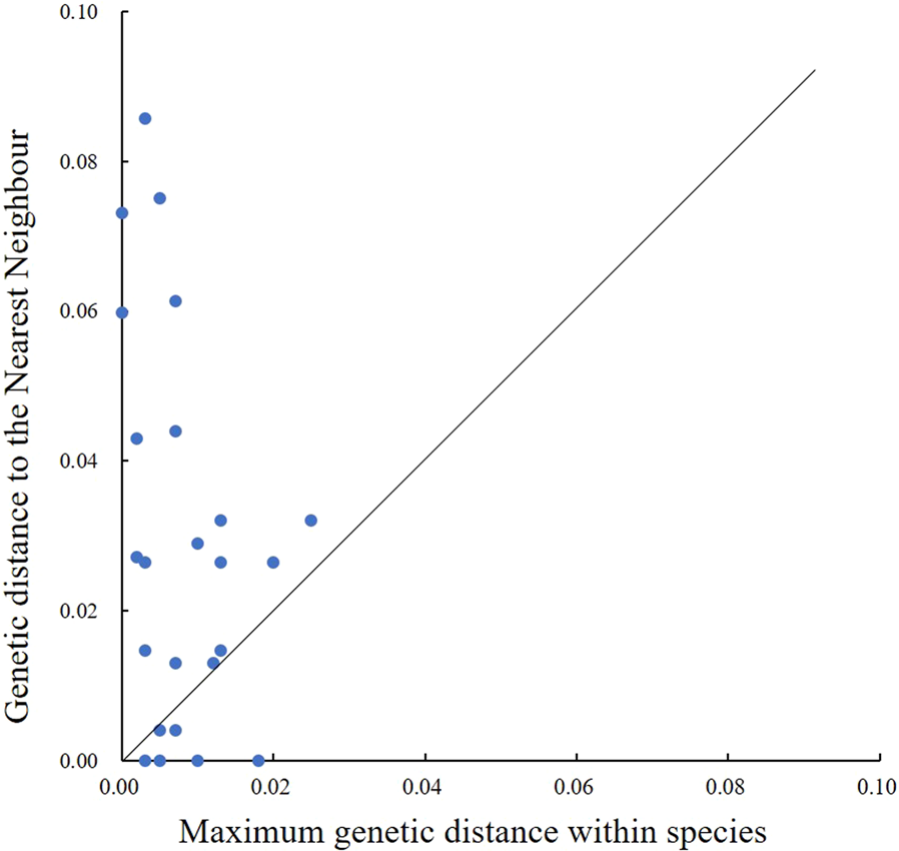
Relationship between the maximum genetic distance within species and nearest neighbour genetic distance among species

The average Kimura 2-parameter (K2P) intraspecific distance ranged between 0 and 3.10% (Table 2). The maximum observed average K2P intraspecific distance was that of *T. robusta*. The maximum intraspecific K2P distance ranged from 0 to 7.90%. The largest K2P intraspecific distance was observed for *T. robusta*, followed by *T. minxianensis*, with a value of 7.40%. The nearest neighbour distance ranged between 0 and 8.57%. For *T. robusta*, *T. minxanensis*, *T. siluroides* and *T. pappenheimi*, a nearest neighbour distance of 0% was observed. The nearest neighbour distance of 18 species was lower than the maximum K2P intraspecific distance. Only the nearest neighbour distance of *T. scleroptera* and *T. pseudoscleroptera* was less than 1%, at 0.40%. The distributions of the maximum K2P intraspecific distances and the nearest neighbour K2P genetic distances reflected the overlap; in addition, no barcode gap was found (Fig. 4).

**Table 2:** Genetic K2P distances of the *Triplophysa* species

Species	MOTU	Mean intra-	Maximum intra-	NN Dist	NN
*Triplophysa bleekeri*	MOTU-1	0.0000	0.0000	0.0731	*T. papillosolabiatus*
*T. shiyangensis*	MOTU-3	0.0069	0.0130	0.0265	*T. stoliczkae*
*T. strauchii*	MOTU-4	0.0017	0.0020	0.0271	*T. stoliczkae*
*T. orientalis*	MOTU-5	0.0000	0.0000	0.0598	*T. stoliczkae*
*T. tenuis*	MOTU-7	0.0039	0.0070	0.0613	*T. pseudoscleroptera*
*T. wuweiensis*	MOTU-8	0.0033	0.0050	0.0751	*T. obscura*
*T. sp2*	MOTU-9	0.0028	0.0030	0.0264	*T. obscura*
*T. obscura*	MOTU-10	0.0101	0.0200	0.0264	*T. sp2*
*T. polyfasciata*	MOTU-11	0.0022	0.0030	0.0857	*T. bleekeri*
*T. sp1*	MOTU-12	0.0099	0.0100	0.0290	*T. papillosolabiatus*
*T. leptosoma*	MOTU-13	0.0022	0.0030	0.0147	*T. papillosolabiatus*
*T. papillosolabiatus*	MOTU-14	0.0053	0.0130	0.0147	*T. leptosoma*
*T. sellaefer*	MOTU-16	0.0036	0.0070	0.0440	*T. minxianensis* sp1
*T. hsutschouensis*	MOTU-18	0.0063	0.0130	0.0320	*T. robusta*
*T. scleroptera*	MOTU-6	0.0030	0.0050	0.0040	*T. pseudoscleroptera*
*T. pseudoscleroptera*	MOTU-6	0.0040	0.0070	0.0040	*T. scleroptera*
*T. stoliczkae*	MOTU-2	0.0060	0.0120	0.0130	*T. dalaica*
*T. dalaica*	MOUT-2	0.0040	0.0070	0.0130	*T. stoliczkae*
*T. robusta*		0.0310	0.0790		
	MOTU-19	0.0082	0.0250	0.0320	*T. hsutschouensis*
	MOTU-15	0.0077	0.0180	0.0000	*T. pappenheimi, T. siluroides*
*T. minxianensis*		0.0160	0.0740	0.0110	*T. siluroides*
	MOTU-17	0.0016	0.0020	0.0430	*T. robusta*
	MOTU-15	0.0050	0.0100	0.0000	*T. siluroides*, *T. pappenheimi*
*T. siluroides*	MOTU-15	0.0030	0.0050	0.0000	*T. pappenheimi*, *T. robusta*
*T. pappenheimi*	MOTU-15	0.0020	0.0030	0.0000	*T. siluroides*, *T. minxianensis*

The mean and the maximum of intra-group distances, the nearest neighbor (NN), and the minimum distance to the NN for the Nominal species

**Fig. 5:**
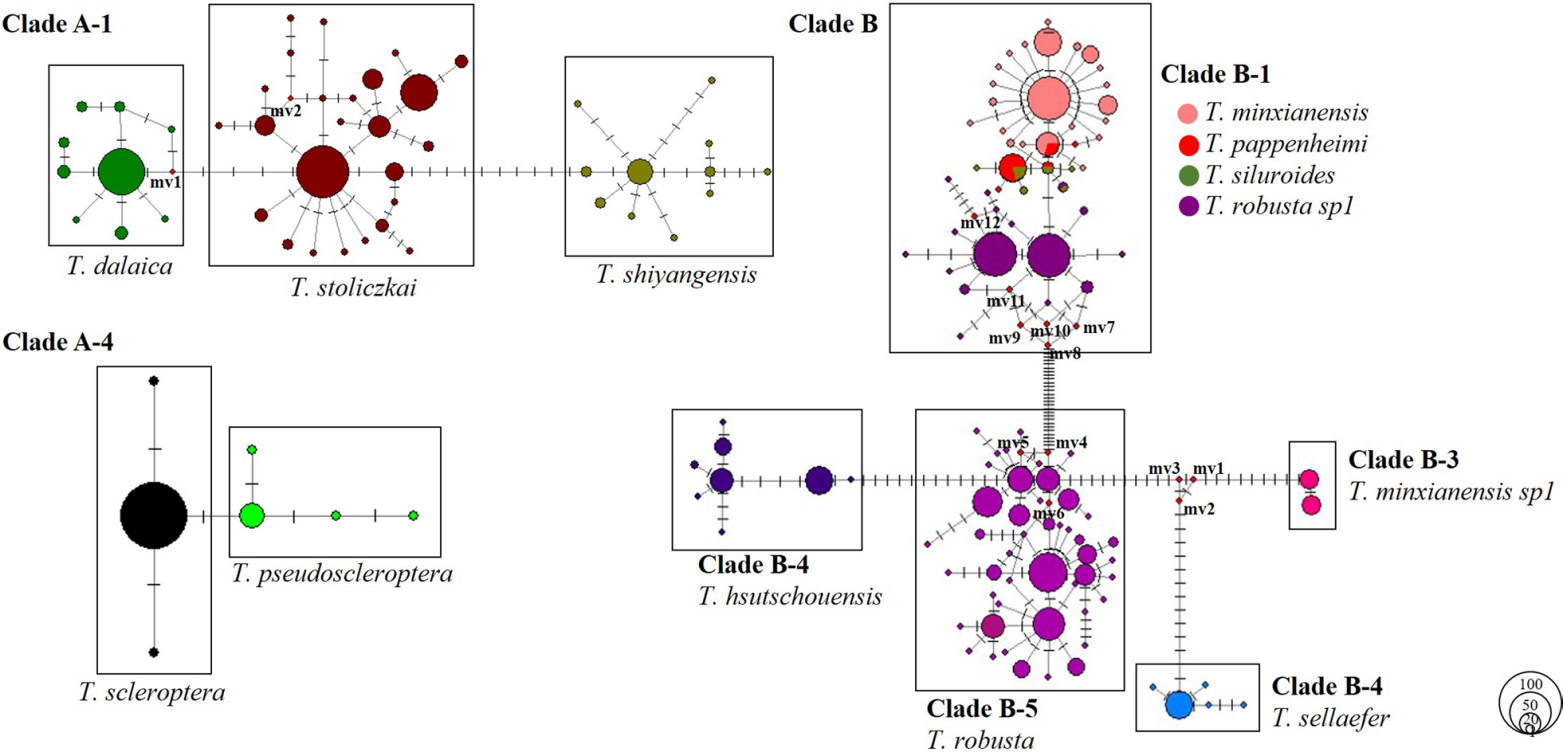
Haplotype networks for the species group involved in mixed genealogies. (The area of circles is proportional to the haplotype frequencies, and mv1–mv12 are missing haplotypes. Lines linking haplotypes indicate the evolutionary paths among haplotypes, and vertical bars on the linking lines represent the mutation steps between haplotypes.)

Most species form very good evolutionary clades in the NJ tree, and these main clades represent different taxonomic species. Monophyletic clades were also observed for *T. stoliczkae* and *T. dalaica*, and *T. scleroptera* and *T. pseudoscleroptera*. Neither *T. minxianensis* nor *T. robusta* formed an independent monophyletic clade; however, they formed two larger clades according to geographic distribution. Because of the shared haplotype among *T. minxianensis*, *T. pappenheimi*, *T. siluroides* and *T. robusta*, these four species formed a larger clade. The trend of mixed genealogies was confirmed by the examination of the haplotype networks. Two species pairs (*T. stoliczkae* and *T. dalaica* (Fig. 5 clade A-1) and *T. scleroptera* and *T. pseudoscleroptera* (Fig. 5 clade A-4)) could not be distinguished by the four algorithms used for MOTU delimitation, and haplotypes were not shared between them. Four haplotypes were shared among *T. minxianensis*, *T. pappenheimi*, *T. siluroides* and *T. robusta* (Fig. 5 clade B).

## Discussion

In this study, a total of 24 species were reported, including two new species: a cryptic species in the *T. minxianensis* population and a cryptic species in the *T. robusta* population. The morphological and molecular data were consistent in 14 of the 22 species identified. The results showed that two cryptic species could be described in the biodiversity hotspot area, which reinforces the general view that a large amount of unrecorded diversity remains in the plateau loach. For example, only one haplotype has been identified in the clade of *T. bleekeri* and *T. orientalis*. Therefore, more specimens must be collected, sequences must be added, and the possibility of identifying more cryptic species cannot be ruled out.

Different numbers of MOTUs were identified in the four DNA barcode analysis methods: 17 different MOTUs were identified using the PTP and GMYC models, and 19 MOTUs were identified using the ABGD and BOLD methods. *T. shiyangensis* and *T. leptosoma* could not be distinguished by the PTP or GMYC model; however, the ABGD and BOLD methods allowed different MOTUs to be assigned to each species (Fig. 3). The inconsistent results of the four methods may be due to differences in the methods used to distinguish species. The ABGD and BOLD methods are based on the genetic distance between species and distinguish species by the difference of intraspecific and interspecific genetic distances. The BOLD method defaults to a genetic distance threshold of 2.2% and ABGD of 2.8%, resulting in the same number of MOTUs defined by the two methods. Although the RESL in the BOLD system has a stronger taxonomic performance than that in the ABGD system and thus shows better species identification and MOTU assignment results [59], the two methods in this study achieved the same results, which may be related to the identified species. A key aspect implicit in DNA barcoding analysis is the genetic distance threshold values used to define the MOTUs. COI genetic distance values from 1 [30] to 2% [33] have been considered the threshold values for fish DNA barcoding analysis. However, these values are derived from comparative analyses of species diversity in different aquatic ecosystems. For example, 2% is used to represent the DNA barcodes for the community of fish in certain rivers [51]. However, when DNA barcoding analysis was used for a group of closely related species (e.g., the same genus), a lower genetic distance value has been reported [10, 51, 52]. In particular, a low threshold value of 0.92% is needed to distinguish MOTUs in the genus *Laemolyta* (Anostomidae) [58]. Although most of the values obtained in this paper are above 1.47% (14 of 18 MOTUs, Table 2), the maximum threshold value of related species detected between the obtained MOTUs is 0.40%, and some species have shared haplotypes. The existence of haplotype sharing among different species of plateau fishes may be related to complex species differentiation mechanisms or convergent evolution associated with local adaptation [13, 65]. A lower threshold of genetic distance may be obtained when the genetic relationships between different species within a genus are analysed. Although this approach based on genetic distance analysis is easy to perform, it lacks phylogenetic content, uses artificial boundaries to distinguish species and lacks the objectivity of species evolution [48]. The GMYC and PTP methods define species based on evolutionary trees. GMYC uses ultrametric trees to define species [23], and PTP uses substitution calibrated trees to define species, which avoids the potential pitfalls of constructing time-calibrated species phylogeny [80]. We believe that tree-based techniques are effective in identifying individual species because identifying a particular branch representing a particular species requires a threshold to represent the clade length and/or the pair distance used to distinguish differences between individuals [26, 43, 71]. Such thresholds are also required when DNA barcoding data are analysed using clustering methods and based on distance methods. A technical problem with clustering is that paired distances of three or more sequences need not be equal,therefore, strict thresholds are usually impossible to apply [47]. Both methods defined 17 MOTUs in this study. Clearly, the accuracy of DNA barcoding methods depends largely on the target species being analysed [50].

The difference in the number of MOTUs detected by the different analysis methods was mainly seen in two pairs of MOTUs, with relatively low genetic distance observed between *T. shiyangensis* and *T. stoliczkae* (2.65%) and between *T. leptosoma* and *T. papilloso-labiatus* (1.47%). These relatively low genetic distance values may be related to the late differentiation of these MOTUs. Notably, the MOTUs of relatively recent origin had less time than species of distant origin to accumulate genetic differences, which hindered their correct identification, despite the species differing greatly in their morphological characteristics. *T. papilloso-labiatus* has an obvious swim bladder, while *T. leptosoma* does not [82]. The characteristics of the genetic diversity of these species are the same, and they both show a relatively high level of haplotype diversity (> 0.5) and a relatively low level of nucleotide diversity (< 0.5%) (Table 1). These characteristics indicate that after the differentiation of these species, the founder effect and environmental heterogeneity caused by water system changes led to the rapid accumulation of variation in the population, resulting in a high haplotype diversity index. The accumulation time of the nucleotide diversity index was much longer than that of the haplotype diversity index. In terms of geographical distribution, these two species are mainly distributed in the Shulehe River and Heihe River. The possibility of sympatric speciation exists; however, this supposition needs to be confirmed by further analysis.

An example of incompletely separated species was also found. *T. minxianensis*, *T. robusta*, *T. pappenheimi* and *T. siluroides* are not sufficiently differentiated by COI gene differences, and there are also shared haplotypes among the four species (Fig. 5). These phenomena can be explained as frequent mitochondrial DNA introgression events before species differentiation [21] or phenotypic plasticity in fish [61, 68]. The morphological characteristics of *T. hsutschouensis,* which was identified as an independent species isolated from *T. robusta*, include bare and scaleless bodies and a relatively low ratio of body length to body height [73]. *T. robusta* only has residual scales in specific parts of its body. The Jinghe River population of *T. robusta* has scales along the lateral line from the caudal fin to the front of the dorsal fin. Moreover, the Jinghe River population and other populations of *T. robusta* were clustered into two clades (Fig. 3), and the genetic distance between the populations reached 7.9% (Table 2). These phenomena suggest the existence of cryptic species of *T. robusta*. Differences were not observed between *T. minxianensis* and *T. minxianensis sp1* in the degradation of the swim bladder, whether the end of the pelvic fin reached the anus, the starting point of the dorsal fin and the pelvic fin relative to each other or the morphological measurement data. However, the scales of *T. minxianensis sp1* were only found in the caudal peduncle, which is quite different from the scale pattern of *T. minxianensis*, in which all the body parts except the head have obvious round scales. The genetic distance between the two populations was 7.4% (Table 2), which indicated that cryptic species occurred in *T. minxianensis*. Similar to this example of incomplete species separation, Wang [73] argued that the plateau loach groups without scales (*T. hsutschouensis*) come from scaly groups (*T. minxianensis*) following the degeneration of scales. The groups with remnant body scales (*T. robusta*) are the intermediate species between the two types. The presence or absence of scales marks a leap in the evolution of plateau loach populations. The cryptic species found in this study provide more evidence for this speculation.

The morphological characteristics and molecular characteristics were inconsistent in *T. pseudoscleroptera* and *T. scleroptera*. The two species have similar appearances but different internal anatomical structures. The anterior and posterior segments of the swim bladder of *T. pseudoscleroptera* are the same size, with a long pouch or oblong oval shape and no pyloric caecum. The posterior chamber of the swim bladder of *T. scleroptera* is developed, the anterior segment is thin, and the posterior segment is enlarged into a long pouch [83]. Without the comparison of internal anatomical structure, these species are easy to misidentify, and morphological identification may be incorrect (He et al. 2008). However, due to the low interspecific distance between the two species (0.40%), the two MOTUs could not be correctly distinguished. This inconsistency was also found between *T. dalaica* and *T. stoliczkai*. The posterior chamber of *T. dalaica*’s swim bladder was oval, while the posterior chamber of *T. stoliczkai*’s swim bladder was degraded; thus, this feature can be used to accurately distinguish the two species.

As shown by the two cases reported here, DNA barcoding did not identify enough differences to distinguish similar species because the lineages were not completely divided into different clades. The reason for this phenomenon is the process of incomplete lineage sorting. Due to the extremely short time of species differentiation, ancestral traits are randomly fixed in the differentiated species [22, 38]. Similar phenomena have been found in *Psorophora* [12], Syngnathidae [78] and *Laemolyta* [58], and mixed lineage cases are particularly common in plateau fish [65]. In this sense, to find evidence of reproductive isolation, nuclear genetic and ecological data must be combined for further research [6, 45, 70].

Species with morphological characteristics that are not significantly different may be easily identified as a single species. For example, *T. bleekeri* and *T. polyfasciata* have very similar morphological characteristics and do not present significant differences in quantitative traits in different proportions of their bodies, and they have been identified as the same nominal species. Ding et al. [17] believed that they should be divided into two different species based on molecular data and pointed out that the main distinguishing feature was that 10–12 wide dark brown horizontal stripes occurred on the side of the body. However, even among *T. bleekeri* individuals collected from the same site, the number of horizontal stripes on the side of its body can range from 0 to 10. Of the specimens collected from the Wenchuanhe River in Sichuan Province, most had 5–7 horizontal stripes, and almost none had more than 10. Therefore, the validity of *T. polyfasciata* is still questionable (He et al. 2008). In this study, the numbers of these two species of plateau loach collected were relatively small, with 10 T*. bleekeri* and 5 T*. polyfasciata*, and 7–9 horizontal stripes were observed on the sides of the fish bodies. Although the division into two different species was also not supported by morphology, the genetic distance between the two species reached 8.57%, which far exceeded the threshold of genetic distance within the species of 2% [51]. Therefore, these two species likely underwent genetic differentiation in terms of genetic material; however, due to the small size (the length of the collected sample is 5–8 cm), the morphological difference is not obvious; therefore, they have historically been regarded as one species. Obviously, the body colour or body markings of plateau loach may not be an effective classification feature for the identification of species and cannot be used as the main basis for identification.

Herzenstein [29] identified *T. papilloso-labiatus* as a subspecies of *T. strauchii*, and this finding was also supported by Zugmeyer [85]. *T. strauchii* lack a developed mastoid process similar to that of *T. papilloso-labiatus* and only have a strong naked fold; however, the mastoid process on the upper lip of plateau loach inhabiting the Hexi Corridor is obviously a double line, while that on the lower lip is a blurred double line. Characteristics such as the mastoid process and a strong naked crease are continuously transitive in a geographical distribution without obvious boundaries. However, the appearance of significant double lines on the mastoid marks discontinuity in the variation, and relatively stable differences are also observed in a series of other morphological traits. Thus, *T. papilloso-labiatus* should be regarded as an independent species [41, 82], which is also supported in the phylogenetic tree constructed in this study (Fig. 3). *T. strauchii* and *T. papilloso-labiatus* are clustered into two different clades and should be independent species.

Limited differences are observed in the morphological characteristics between *T. wuweiensis* and *T. scleroptera*. Li and Chang [41] regarded *T. wuweiensis* as an independent species based on 7 morphological traits. Zhu and Wu [83, 84] believed that a certain continuity occurs in the identification characteristics of these two species. However, after collecting specimens of *T. scleroptera* distributed in the Datonghe River, which is only one mountain away from the collection location of the *T. wuweiensis* specimens, Zhao [82] believed that significant differences occurred between the two species in the number of pectoral fin rays, intestinal shapes and gill rakers, thus supporting *T. wuweiensis* as an independent species. In this study, *T. wuweiensis* and *T. scleroptera* clustered into different clades, and the two species were greatly differentiated, which also support the idea that *T. wuweiensis* is an independent species. The low genetic diversity of *T. wuweiensis* may be due to the short time since species differentiation and the low haplotype diversity, and nucleotide diversity may be caused by the founder effect and the narrow distribution area (the species is distributed only in the east and west Shiyanghe River tributaries).

*T. shiyangensis*, *T. papilloso-labiatus* and *T. hsutschouensis* are distributed in three inland river systems in the Hexi Corridor. The maximum intra-species genetic distance of these three species is more than 1%, which may be mainly due to the wide geographic distribution of the three species and the large population differentiation caused by the barriers created by the water systems. This phenomenon also appears in the sympatric distribution of *Gymnocypris chilianensis*, in which each geographic population is clustered into a single clade with large genetic differentiation [81].

The different geographic populations of some widespread species are identified as different species or subspecies due to some more significant morphological differences. For example, *T. stoliczkae* was divided into 7 subspecies [29] due to the differences in the number of gill rakers, the proportion of quantitative traits and the number of spiral loops of intestinal tubes with changes in altitude or water system. In this study, the samples were collected in three drainage systems (the Yellow River, Jialing River and inland rivers in the Hexi Corridor). The maximum genetic distance within the species was greater than 1.2% (Table 2). However, the samples of different water systems have shared haplotypes, which indicates that different geographic populations of *T. stoliczkae* in the surveyed area are from a common ancestor.

The membranous swim bladder of *T. obscura* is very developed, with a constriction in the middle, and its length accounts for approximately 2/3 of the abdominal cavity. In contrast to *T. orientalis*, its body surface has obvious spines and is regarded as an independent new species [39]. In this study, a relatively large number of samples (n = 234) were collected in the distribution area. The phylogenetic tree showed that the samples from different water systems were clustered into different clades, the maximum genetic distance within the species was 2%, and the nucleotide diversity and haplotype diversity were relatively high (h = 0.887, π = 0.00777). These findings indicate that large differentiation occurs between the two geographically separated populations of *T. obscura* and the possibility of allopatric speciation. *T. obscura* and *T. orientalis* were also divided into two different monophyletic lines in the phylogenetic tree, which is consistent with the results of the analysis of Wu [77].

Although only 3 specimens of *T. sp1* were collected in the Liangdang section of the Jialing River, obvious differences were observed in morphological characteristics from other species of plateau loach. Thus, it should be identified as a new species that has not been reported; in addition, more specimens should be collected for further confirmation. *T. sp2* was collected in the Jialing River and showed degeneration of the membranous swim bladder, leaving only a small chamber, an anus near the start of the anal fin, the end of the pelvic fin adjacent to the anus, a large spot on the back of the body, a spot on the side of the body and other morphological characteristics that were obviously different from those of the closely related species *T. obscura*. A detailed description of these newly discovered species is necessary to record the relationship between morphology and molecular identification criteria [70].

## Conclusions

This study is the first comprehensive assessment of plateau loach species in a biodiversity hotspot using standard DNA barcoding. A high-density sample collection was carried out in this area to collect all known nominal species of plateau loach in this region. Although 14 of the 24 taxonomic species can be easily identified by DNA barcoding and classical morphological classification, 10 species pose serious challenges to standardized and automated molecular identification via mitochondrial DNA. Newly discovered species and cryptic species identified through DNA barcoding technology reveal the need for a taxonomic revision of the genus. If combined with the MOTUs identified here, the study of morphological features can be facilitated to support the delimitation of species and classification. Moreover, using just one standardized barcode gene is not a perfect method of identifying species because their lower nucleotide sequence differences may be ignored [59]. Therefore, nuclear markers must be combined with ecological and biological data [2, 18, 72], and the survey area and number of species must be expanded to evaluate the species boundary of the plateau loach genus. This study provides a basis for protecting the biodiversity of plateau loach.

## Methods

### Sample collection

The samples were collected at 114 sampling sites in two exorheic rivers (the Jialing River, which is the largest clade of the Yangtze River, and upstream of the Yellow River) and three inland water bodies (the Shiyanghe River, Heihe River and Shulehe River) located on the northeastern edge of the QTP from 2015 to 2018 (Figs. 1, 2). The specimens were caught using gill nets and cage nets. And they were anesthetized with the concentrations (35 mg/ml) of MS222 (a kind of aquatic animal anesthetic with main chemical composition for 3-Aminobenzoic acid ethyl ester methanesulfonate) (Green HX, Beijing, China). To accurately identify the fish based on taxonomic books, the fresh specimens were examined for specific morphological characteristics [73, 76, 84]. The muscle tissue of each specimen was preserved in 95% ethanol for DNA extraction, and the voucher specimens were stored in 10% formaldehyde solution for further examination of specific morphological characteristics (Additioanl file 1: Table S1).

### DNA extraction, amplification and sequencing

Total genomic DNA was extracted from the muscle tissue using the high-salt method, and a segment of 651 bp from the COI gene was amplified using the published primers FishF1 (5′ TCAACCAACCACAAAGACATTGGCAC3′) and FishR1 (5′ TAGACTTCTGGGTGGCCAAAGAATCA3′) [75]. The PCR amplifications were performed in a volume of 30 μL, which included 21.25 μL of molecular-grade water, 3.0 μL of 10 × PCR buffer, 1.5 μL of each primer (10 mM), 1.5 μL of dNTPs (10 mM), 0.375 μL of Taq polymerase, and 1 μL of template DNA. The PCR conditions consisted of initial denaturation at 94 °C for 5 min; followed by 30 cycles of denaturation at 94 °C for 30 s, annealing at 53 °C for 30 s, extension at 72 °C for 1 min; a final extension step at 72 °C for 10 min, and then a hold at 4 °C. The PCR products were analysed in 1% agarose gels containing ethidium bromide stain and bidirectionally sequenced using sequencing primers. The purified PCR products were sequenced on an ABI 3730 XL DNA System.

### Genetic distance analyses

The sequencing chromatograms were checked by Chromas 1.45 software, and the forward and reverse sequences were assembled and edited with the SeqMan program (DNASTAR Inc., WI, USA). The sequences of each specimen generated in this study were compared and aligned using the ClustalW program. Haplotype number, haplotype diversity and nucleotide diversity were calculated with DnaSP 5.0 [42]. A NJ tree and intraspecific and interspecific genetic distances were constructed based on the Kimura 2-parameter (K2P) distance model using MEGA version 5.0 with the bootstrap support values calculated with 1000 replicates [66]. Mr Bayes 3.2.5 software was used for the BI analysis [62]. The posterior probability represents the credibility of each clade. In the BI method, the Bayesian analysis based on codon partitions (1st, 2nd, and 3rd) was used, the random tree was taken as the starting tree, and the GTR + G substitution model was applied. The MCMC length was 50 million generations, with samples saved every 500 generations. The first 10% of trees was discarded as burn-in. The average standard deviation of split frequencies (ASDSF < 0.01) was used to assess the criteria of convergence, and the final ASDSF was 0.006. The ML method was analysed with PhyML 3.0 software [25], the substitution model was defined as GTR + G, and the number of substitution rate categories was set as 6. The best-scoring ML tree was determined by 100 repetitions. Then, a bootstrap analysis was performed 1000 times to estimate the bootstrap support of the nodes. These values were used to calculate the maximum, minimum and mean intraspecific and interspecific MOTU distances [57]. The average K2P intraspecific distance was defined as the average within each nominal species, the maximum K2P intraspecific distance was defined as the largest distance observed between sequence pairs within a nominal species, and the barcoding gap was checked by the intraspecific and interspecific genetic distances [65]. Finally, we constructed haplotype networks for mixed genealogies species (or main clades B) using Network 4.6 software [3].

### Species delimitation

The species identified based on morphological characteristics were referred to as valid species, and species delimited by DNA sequences were referred to as MOTUs [31, 65]. Four MOTU delimitation algorithms were used to delimit species. Two analysis methods based on genetic distance and two analysis methods based on evolutionary tree topology were used to classify the molecular clades or MOTUs. Among them, the BOLD and ABGD are two analysis methods based on genetic distance, and the PTP and GMYC are two analysis methods based on evolutionary tree topology. The barcode index number (BIN) system was used to delimit MOTUs automatically in the BOLD workbench (https://v4.boldsystems.org/) [59]. The sequencing data were uploaded to BOLD Systems, the cluster sequences in the sequence analysis were used to obtain the MOTUs for the whole sequence, and then a Taxon ID Tree in the sequence analysis was used to generate evolutionary tree delimitation species. The ABGD was used via a web interface (https://wwwabi.snv.jussieu.fr/public/abgd/abgdweb.html) [56] and is an online analysis software that automatically searches barcode gap location and classifies sequences into assumed species. The following parameter settings were applied: Pmin was set to 0.001, Pmax was set to 0.1, Steps was set to 10, X was set to 1.5, and Nb bins were set to 20. A partition result of P = 0.0278 was selected as the final ABGD partition result.

A PTP was used to delimit the species through the bPTP server (https://species.h-its.org/ptp/), including a Bayesian likelihood PTP with an ML partition and Bayesian implementation [80]. ModelFinder in PhySuite was used to select the best Bayesian tree model [32]. The *.fas file was converted into a *.nex file by EasyCodeML [24], and the corresponding optimal model and corresponding parameters were set. The parameters were set to 10 million generations; sampling was performed once every 100 generations; and the outgroup was set. The *.nex file was input into MrBayes to build the BI evolutionary tree. The BI tree parameters were checked, and when the ASDSF was less than 0.01, then the results of the two runs had little difference, which indicated that the parameters converged. After merging the two *.p files, all the effective sample size (ESS) statistical parameters were greater than 200, indicating that the parameters converged. Then, Fig Tree V1.4.4 (https://tree.bio.ed.ac.uk/software/figtree/) was used to view the obtained BI tree and convert it to Newick format. The tree was uploaded to the online bPTP, the root tree was set, the external group was removed, the number of reversible Markov chains was set to 500,000, and other parameters were set to default for analysis.

The GMYC model in the R package Splits 1.0-19 [23] was used to infer the MOTUs. ModelFinder in PhySuite was used to select the nucleotide replacement model, and the hypermetric tree was constructed based on Software Beast 2.4.8 (Bouckaert et al. 2014). The assumption of a strict molecular clock was set, and the system generation model was a birth–death process and a GTR + G substitution model. The MCMC chain was 10 million generations, sampling was performed once every 10 thousand generations, and the first 10% of trees was discarded as burn-in. Tracer 1.6 was used to evaluate the convergence of the system tree and check the *.log.txt file. All ESS values were greater than 200, which indicates that the system tree converged. The *.rains.txt file that contained the tree file was imported into TreeAnnotator v1.8.2, burn-in was set to 1000, and the maximum clade credibility tree was generated. Fig Tree V1.4.4 was used to convert the tree file to Newick format, and the R package Splits 1.0–19 was used to analyse the GMYC.

## Supplementary information

**Additional file 1.** Checklist for plateau loach, including their distribution and GenBank accession numbers.

## Data Availability

Table S1 Checklist for plateau loach, including their distribution and GenBank accession numbers.

## Abbreviations

QTP: Qinghai-Tibet Plateau
COI: Mitochondrial cytochrome C oxidase subunit I
BOLD: Barcode of life data systems
NJ: Neighbour-joining
ML: Maximum likelihood
BI: Bayesian inference
K2P: Kimura 2-parameter
ASDSF: Average standard deviation of split frequencies
MOTU: Molecular operational taxonomic unit
PTP: Poisson tree processes
GMYC: General mixed Yule-coalescent
BIN: Barcode Index Number
ABGD: Automatic barcode gap discovery
